# Effects of a PSEN2 isogenic series on autophagy and Abeta production in iPSC‐derived neurons

**DOI:** 10.1002/alz70861_108908

**Published:** 2025-12-23

**Authors:** Adriana Cunha, Tatiana A. Giovannucci, Mateo Barro Fernandez, Gustavo Parfitt, Ross W. Paterson, Charlie Arber, Selina Wray

**Affiliations:** ^1^ University College London, London, London UK; ^2^ Department of Neurodegenerative Disease, UCL Institute of Neurology, Queen Square, London UK; ^3^ University College London, WC1N 1PJ, London UK; ^4^ Dementia Research Centre, Institute of Neurology, University College London, London UK; ^5^ Darent Valley Hospital, Dartford UK; ^6^ Dementia Research Centre, UCL Queen Square Institute of Neurology, London UK; ^7^ UCL Queen Square Institute of Neurology, London UK

## Abstract

**Background:**

PSEN1 and PSEN2 are catalytic subunits of transmembrane gamma‐secretase complexes, which along with beta‐secretase in the amyloidogenic pathway, successively cleave APP into Abeta peptides of varying lengths. Mutations in *PSEN1/PSEN2* cause lysosome‐related pathology, along with neuronal loss and amyloid deposition in early‐onset, familial AD (fAD).

**Method:**

We carried out an investigation of autophagy function and Abeta production in an isogenic series of human induced pluripotent stem cell (iPSC)‐derived cortical neurons from the iPSC Neurodegenerative Disease Initiative (iNDI) carrying the PSEN2^N141I^ variant, the most prevalent PSEN2 fAD mutation. We measured autophagic flux via western blotting and imaging of the autophagy‐related proteins LC3B and SQSTM1/p62 in the absence or presence of chemical autophagy modulators. We measured release of Abeta species (Abeta 42, 40, 38) in the media via MesoScale Discovery ELISA.

**Result:**

PSEN2^N141I^ did not affect the pluripotency of the iPSCs nor their potential to differentiate into cortical neurons. As expected, PSEN2^N141I^ increased the AD pathology‐associated Ab42:Ab40 ratio in a stepwise manner, where the homozygous line had a significantly higher ratio than WT (P < 0.01) but comparatively less pronounced than PSEN1 mutants (homozygous int4del) and distinct to their corresponding knockouts. Heterozygous PSEN2^N141I^ cells showed no defect in autophagy readouts, while PSEN2 N141I/N141I cells showed significantly enhanced autophagic flux compared to WT. An analogous trend was also observed for ubiquitin flux and at the transcriptional level, with TFEB and cathepsin D transcript upregulation in PSEN2 N141I/N141I cells.

**Conclusion:**

These data show PSEN2 mutants have decreased gamma‐secretase processivity, and suggest that mutations in PSEN2 cause imbalances in lysosome‐associated proteostasis in human‐derived neurons which may predispose neurodegeneration associated with age.

